# Supplementary figures and images for: The role of vicariance and dispersal on the temporal range dynamics of forest vipers in the Neotropical region
Source: PLoS One. 2021 Sep 17;16(9):e0257519. doi: 10.1371/journal.pone.0257519 (PMC8448354; doi:10.1371/journal.pone.0257519)

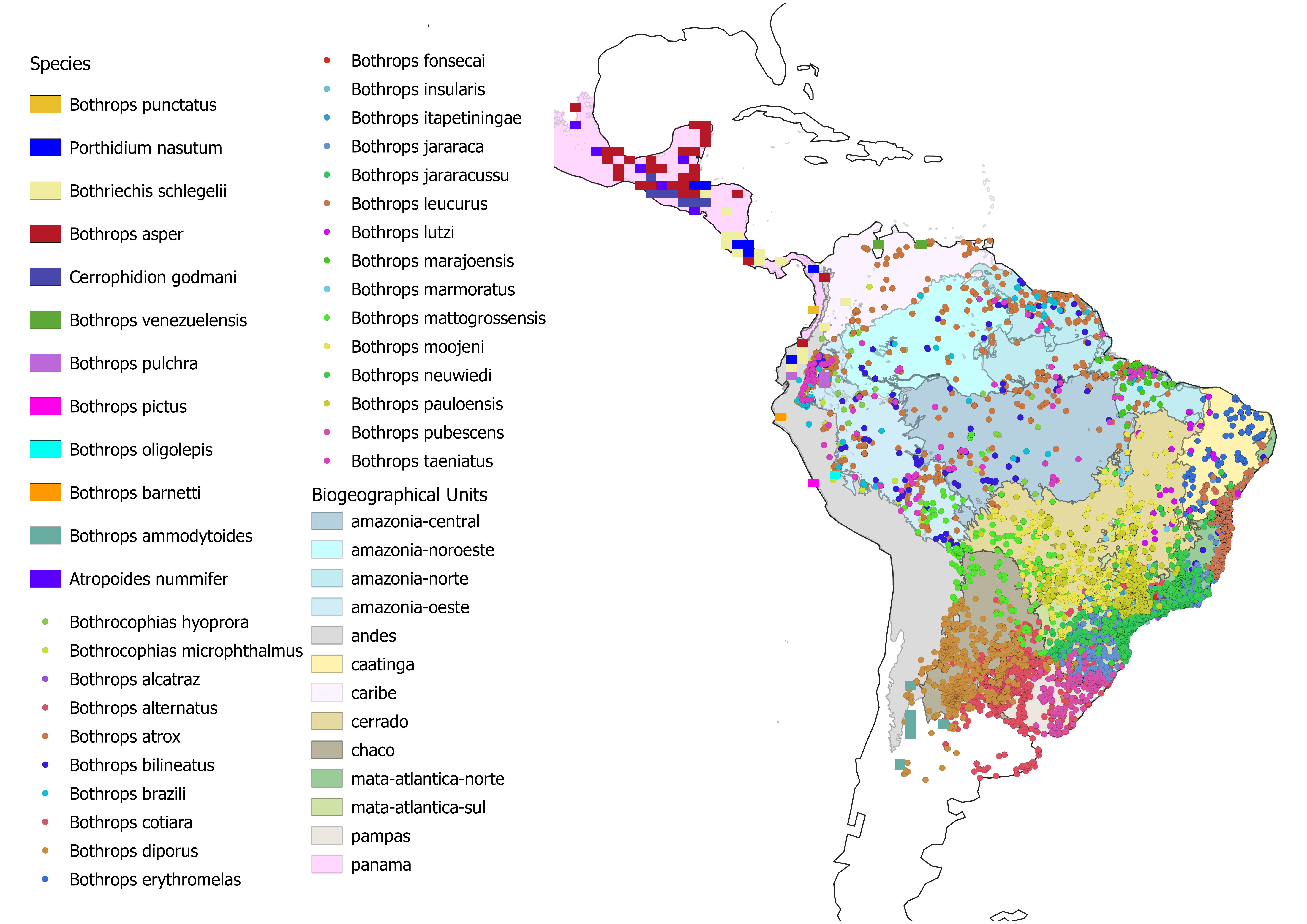

Supplement: S1 Fig — The occurrences indicated by squares are from Guedes et al. [93], and those dots are from Nogueira et al. [51]. The distributions that were recovered from Uetz et al. [56] and Carrasco et al. [58] are not shown, as they are descriptions. The units correspond to the units used in this study (Fig 2). Map made with Natural Earth. Free vector and raster map data from naturalearthdata.com. (TIFF) [file pone.0257519.s005.tiff]

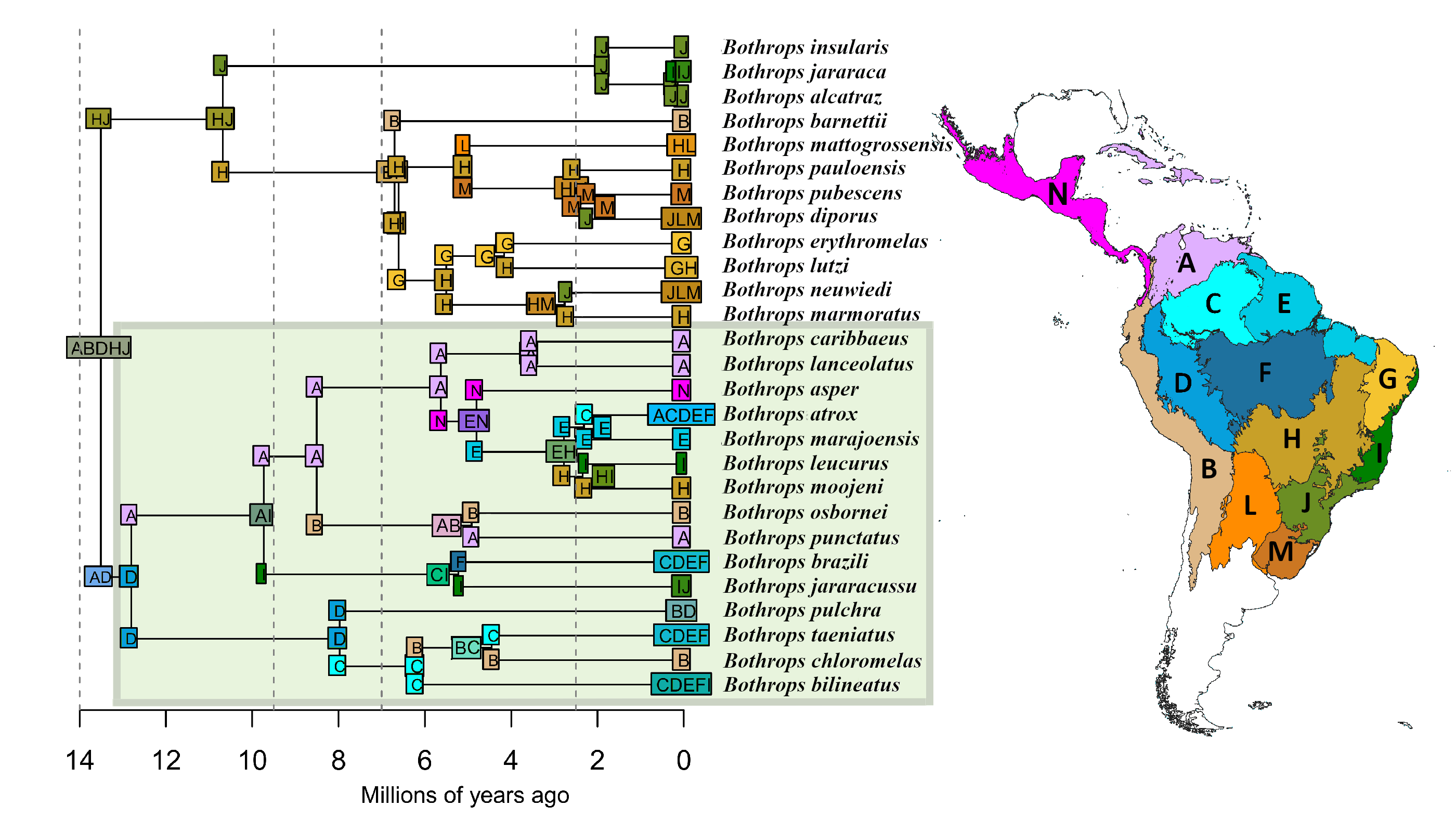

Supplement: S2 Fig — Single capital letters indicate different biogeographical units used in this study. Mixed letters represent combinations of units. Colours also represent biogeographical units. Combinations of two or more units are shown as a mixed colour made from all the units in the combination. Units next to species names represent the current geographical distribution of each species. The green clade showcases the focal forest clade. Vertical dashed gray lines mark the time slices defined in the time stratified matrix. Letters in corners of the cladogram represent the geographical range inherited from the ancestor immediately after a cladogenetic process. (TIFF) [file pone.0257519.s006.TIFF]

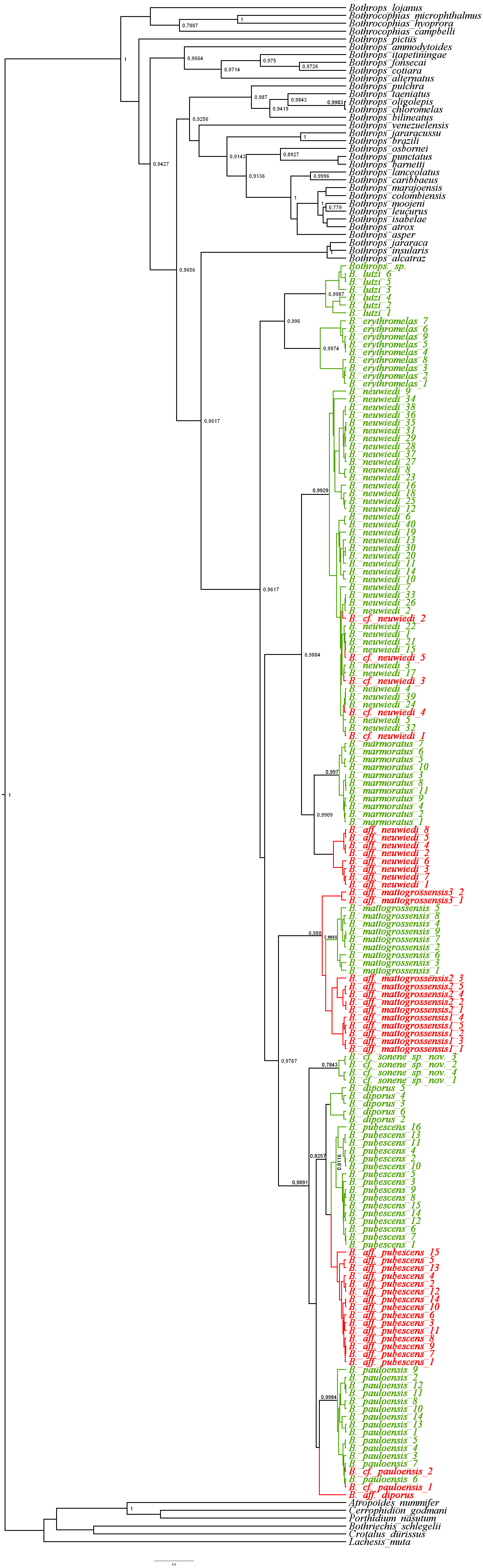

Supplement: S3 Fig — Green clades were collapsed in the final phylogeny. Red clades were removed from the final phylogeny. Posterior probabilities higher than 0.75 are present at nodes (for some internal nodes we also occulted some posteriors for better visualization). (TIFF) [file pone.0257519.s007.TIFF]

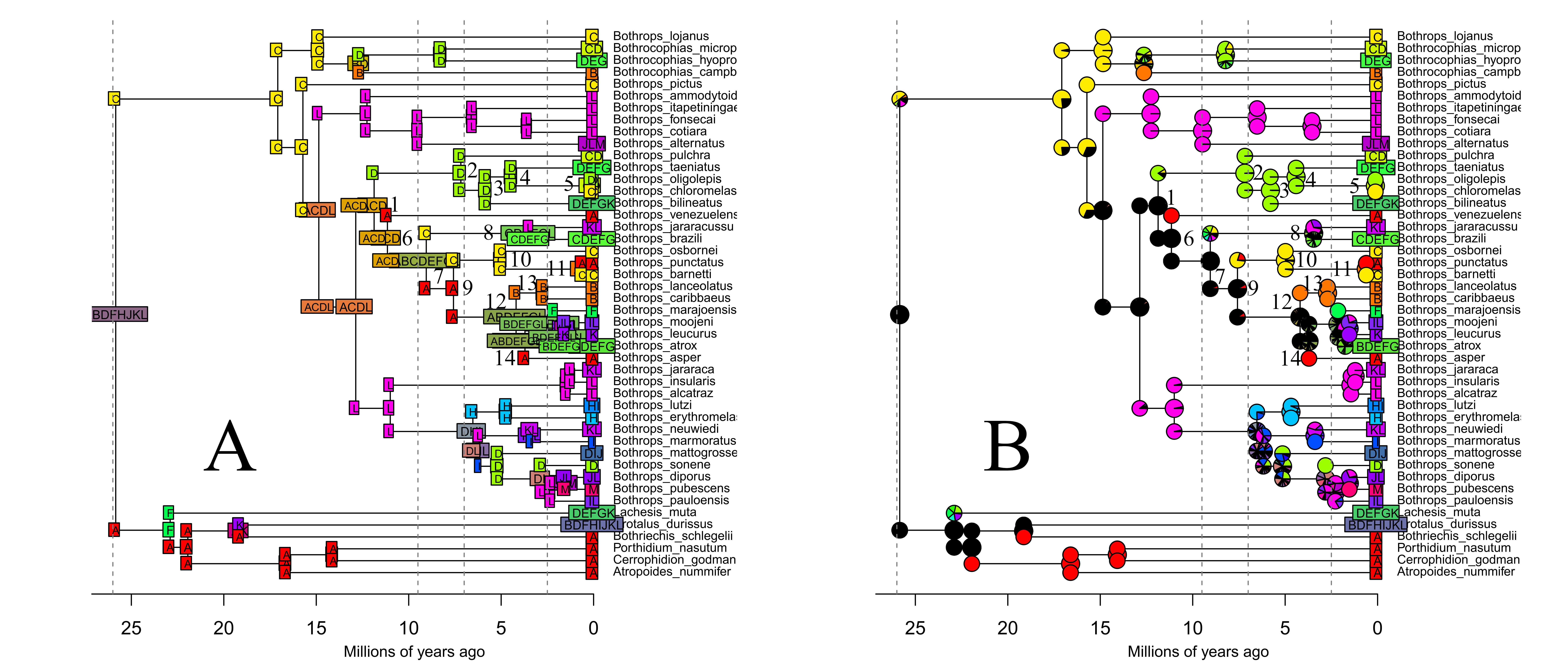

Supplement: S4 Fig — (A) Ancestral geographic ranges reconstructed by the model. (B) Ancestral geographic range probabilities reconstructed by model. Nodes from the forest lanceheads clade are labelled. Single capital letters indicate different biogeographic units used in this study. Mixed letters represent combinations of units.Units next to species names represent the current geographic range of each species. The green clade showcases the focal forest clade. Vertical dashed grey lines mark the time slices defined in the time stratified matrix. Letters in corners of the cladogram represent the geographic range inherited from the ancestor immediately after a cladogenetic process. (TIFF) [file pone.0257519.s008.TIFF]
