# Supplementary material for: The role of vicariance and dispersal on the temporal range dynamics of forest vipers in the Neotropical region
Source: PLoS One. 2021 Sep 17;16(9):e0257519. doi: 10.1371/journal.pone.0257519 (PMC8448354; doi:10.1371/journal.pone.0257519)
Supplement: S1 Table — Examples are from the DIVALIKETS model. The table was heavily inspired and based on Matzke [98]. (DOCX) [file pone.0257519.s009.docx]

| Process | Ranges | | Text | Description | Example |
| --- | --- | --- | --- | --- | --- |
|  | Before | After |  |  |  |
| Dispersal | \| A \| B \| \| --- \| --- \| \| C \| D \|   Ancestor | \| A \| B \| \| --- \| --- \| \| C \| D \|   Ancestor | A → AB | Gain of distribution, a species occurred in only one unit and dispersed into another unit | 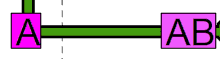 |
| Extinction | \| A \| B \| \| --- \| --- \| \| C \| D \|   Ancestor | \| A \| B \| \| --- \| --- \| \| C \| D \|   Ancestor | AB → A | Distribution loss, a species occurred in two or more units and became extinct locally in one unit | No example |
| Sympatry  (narrow) | \| A \| B \| \| --- \| --- \| \| C \| D \|   Ancestor | Descedant 1   \| A \| B \| \| --- \| --- \| \| C \| D \| \|  \| \| \| A \| B \| \| C \| D \|   Descedant 2 | A → A, A | Two descendants in a cladogenetic process inherit the distribution of the ancestor (single unit) | 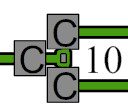 |
| Sympatry  (subset) | \| A \| B \| \| --- \| --- \| \| C \| D \|   Ancestor | Descedant 1   \| A \| B \| \| --- \| --- \| \| C \| D \| \|  \| \| \| A \| B \| \| C \| D \|   Descendant 2 | ABCD → ABCD, A | A descendant in a cladogenetic process inherit the entire distribution of the ancestor, while the other inherits part of it | No example |
| Sympatry  (widespread) | \| A \| B \| \| --- \| --- \| \| C \| D \|   Ancestor | Descendant 1   \| A \| B \| \| --- \| --- \| \| C \| D \| \|  \| \| \| A \| B \| \| C \| D \|   Descendant 2 | ABCD → ABCD, ABCD | Two descendants in a cladogenetic process inherit the entire distribution of the ancestor (two or more units) | No example |
| Vicariance  (narrow) | \| A \| B \| \| --- \| --- \| \| C \| D \|   Ancestor | Descendant 1   \| A \| B \| \| --- \| --- \| \| C \| D \| \|  \| \| \| A \| B \| \| C \| D \|   Descendant 2 | ABCD → A, BCD | Unequal inheritance of the ancestral distribution | 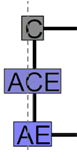 |
| Vicariance  (Widespread) | \| A \| B \| \| --- \| --- \| \| C \| D \|   Ancestor | Descendant 1   \| A \| B \| \| --- \| --- \| \| C \| D \| \|  \| \| \| A \| B \| \| C \| D \|   Descendant 2 | ABCD → AB, CD | Equal inheritance of the ancestral distribution | 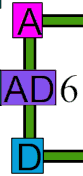 |

This table is heavily based on the Figure 1 from Matzke, 2013 [1].

**References**

1. Matzke NJ. Probabilistic historical biogeography:new models for founder-event speciation, eimperfect detection, and fossils allow improved accurancy and model-testing. Front Biogeogr. 2013;5: 242–248. doi:10.5811/westjem.2011.5.6700
