# Supplementary material for: The role of vicariance and dispersal on the temporal range dynamics of forest vipers in the Neotropical region
Source: PLoS One. 2021 Sep 17;16(9):e0257519. doi: 10.1371/journal.pone.0257519 (PMC8448354; doi:10.1371/journal.pone.0257519)
Supplement: S1 Text — (DOCX) [file pone.0257519.s010.docx]

The Time Stratified Matrix was constructed considering the known landscape evolution of the Neotropical region. The region underwent many transformations over the million years of the evolution of the genus *Bothrops*. These transformations may have interfered in the dispersal rates between the geographical domains explored in our study. The Time Stratified Matrix is a matrix with arbitrary numbers that multiply dispersal probabilities between two regions according to the known landscape evolution, where 1 means total possibility of dispersal between two areas, and 0 means no possibility at all. We divided our matrix into four time slices: the first corresponding to the time frame between the present and 2.5 million years ago (mya); the second between 2.5 and 7 mya; the third between 7 and 9.5 mya; and the fourth between 9.5 and 14 mya.

The first division extends from the present to the beginning of the Pleistocene period (present – 2.5 mya). The Pleistocene period is very important for the Neotropical region as it was during this period that many climatic fluctuations occurred, allowing forest expansions [1–3] which formed corridors connecting both the Atlantic and the Amazon rainforests [3–6].

The second division extends from the beginning of the Pleistocene period to the end of the Acre system (2.5 – 7 mya). In this timeframe, the open diagonal of dry landscapes of the Neotropical region was generated, as the Andes reached their greatest height [7] and cut the air flow from the Pacific ocean.

The third division encompasses the existence of the Acre system (7 – 9.5 mya; [8–10]). In this period, the Amazonas River underwent a change in flow direction resulting in the Acre system. The flow change was an important landscape transformation, as the original flow from the Amazonas River was towards the Pacific [8–10].

The fourth and final division of the matrix begins at the formation of the Acre system up until the beginning of the diversification of the forest lanceheads (9.5 – 14 mya). This timeframe is important as it was when a marine introgression in the northwestern South America created the Pebas system [8–12].

All geographic barriers cited above were considered during the construction of the matrix. When there was a significant geographic barrier between two domains (e.g. the Pebas system or the open diagonal of dry vegetation), we applied a slightly smaller number in the matrix. We also considered the distance between two domains as an important dispersal limitation, so closer domains have slightly high numbers in the matrix. This was done corresponding to the distance, as even though this is not a proper geographic barrier, it may interfere in dispersal rates.

**References**

1. Auler AS, Wang X, Edwards RL, Cheng H, Cristalli PS, Smart PL, et al. Quarternary ecological and geomorphic changes associated with rainfall events in presently semi-arid northeastern Brazil. J Quat Sci. 2004;19: 693–701. doi:10.1002/jqs.876

2. Wang X, Auler AS, Edwards LL, Cheng H, Cristalli PS, Smart PL, et al. Wet periods in northeastern Brazil over the past 210 kyr linked to distant climate anomalies. Nature. 2004;432: 740–743. doi:10.1038/nature03067

3. Oliveira PE, Barreto AMF, Suguio K. Late Pleistocene/Holocene climatic and vegetational history of the Brazilian caatinga: The fossil dunes of the middle Sao Francisco River. Palaeogeogr Palaeoclimatol Palaeoecol. 1999;152: 319–337. doi:10.1016/S0031-0182(99)00061-9

4. Costa LP. The historical bridge between the Amazon and the forest of brazil a study of molecular phylogeography with small mammals. J Biogeogr. 2003;30: 71–86. doi:10.1046/j.1365-2699.2003.00792.x

5. Batalha-Filho H, Fjeldså J, Fabre PH, Miyaki CY. Connections between the Atlantic and the Amazonian forest avifaunas represent distinct historical events. J Ornithol. 2013;154: 41–50. doi:10.1007/s10336-012-0866-7

6. Quijada-Mascareñas JA, Ferguson JE, Pook CE, Salomão MDG, Thorpe RS, Wüster W. Phylogeographic patterns of trans-Amazonian vicariants and Amazonian biogeography: The Neotropical rattlesnake (Crotalus durissus complex) as an example. J Biogeogr. 2007;34: 1296–1312. doi:10.1111/j.1365-2699.2007.01707.x

7. Luebert F, Hilger HH, Weigend M. Diversification in the Andes: Age and origins of South American Heliotropium lineages (Heliotropiaceae, Boraginales). Mol Phylogenet Evol. 2011;61: 90–102. doi:10.1016/j.ympev.2011.06.001

8. Hoorn C, Bogotá-A GR, Romero-Baez M, Lammertsma EI, Flantua SGA, Dantas EL, et al. The Amazon at sea: Onset and stages of the Amazon River from a marine record, with special reference to Neogene plant turnover in the drainage basin. Glob Planet Change. 2017;153: 51–65. doi:10.1016/j.gloplacha.2017.02.005

9. Hoorn C, Wesselingh FP, ter Steege H, Bermudez MA, Mora A, Sevink J, et al. Amazonia Through Time: Andean Uplift, Climate Change, Landscape Evolution, and Biodiversity. Science (80- ). 2010;330: 927–931. doi:10.1126/science.1194585

10. Salas-Gismondi R, Flynn JJ, Baby P, Wesselingh FP, Antoine P-O, Salas-gismondi R. A Miocene hyperdiverse crocodylian community reveals peculiar trophic dynamics in proto-Amazonian mega-wetlands. Proc R Soc B Biol Sci. 2015;282: 4–8. doi:10.1098/rspb.2014.2490

11. Jaramillo CA, Hoorn C, Silva SAF, Leite F, Herrera F, Quiroz L, et al. The Origin of the Modern Amazon Rainforest: Implications of the Palynological and Palaeobotanical Record. Amaz Landsc Species Evol A Look into Past. 2010; 317–334. doi:10.1002/9781444306408.ch19

12. McDermott A. A sea in the Amazon. Proc Natl Acad Sci. 2021;118: 1–6. doi:https://doi.org/10.1073/pnas.2102396118
