# Supplementary material for: The role of vicariance and dispersal on the temporal range dynamics of forest vipers in the Neotropical region
Source: PLoS One. 2021 Sep 17;16(9):e0257519. doi: 10.1371/journal.pone.0257519 (PMC8448354; doi:10.1371/journal.pone.0257519)
Supplement: Attachment — Submitted filename: Response to Reviewers.docx [file pone.0257519.s012.docx]

**Rebuttal Letter**

We are very thankful for the comments and revisions on our revised manuscript made by the Academic editor and reviewer #2. Below is our response to every point raised by the editor and reviewer to improve our work and to fully meet PLOS ONE’s publication criteria. We hope that we satisfyingly addressed these points and that our manuscript will now be suited for publication.

Sincerely,

On behalf of all authors,

Matheus Pontes-Nogueira (corresponding author).

**Academic Editor**

**Journal Requirements:**

**Please review your reference list to ensure that it is complete and correct. If you have cited papers that have been retracted, please include the rationale for doing so in the manuscript text, or remove these references and replace them with relevant current references. Any changes to the reference list should be mentioned in the rebuttal letter that accompanies your revised manuscript. If you need to cite a retracted article, indicate the article’s retracted status in the References list and also include a citation and full reference for the retraction notice.**

Answer: Thanks for this comment. We included 40 new citations in our manuscript in comparison to our original manuscript. However, we unfortunately forgot to mark in the ‘Revised Manuscript with Track Changes' these new references. This newer version has every new citation that we included in the revised manuscript marked accordingly. We further analysed all references to see if they meet PLOS ONE’s citation style and if they are corrected. We hope that now the reference list meets the journal’s criteria.

**Reviewer #2**

**In this new version, Pontes-Nogueira and collaborators have successfully addressed most of my previous comments. There is a great improvement in the quality of the work in all methodological aspects, as well as in the way of transmitting the scientific reasoning, particularly in the introduction. The discussion has also been profoundly modified, now providing a good comparison of the results with the biogeography of different species in the region. My acknowledgements to the authors for such work! I just have a few minor comments:**

Answer: We are very, very thankful for this comment. We are glad to know that we encompassed all the observations that you made in the revision. We hope that once again we fully addressed all your newer comments with this revised manuscript. Thanks once again!

**Line 155 - 159 – please add the number of sequences considered**

Answer: thanks for the comment. We added the number of sequences considered in the text (412 sequences; line 156). Thanks.

**Line 219-220 – please, explain better what you mean here and why you refer to Crotalus durissus**

Answer: We elaborated further this paragraph to explain better what the maximum range size is and why we used the range size of the *C. durissus* in our analysis (lines 219 – 225). We thank you for this advice and comment.

**Lines 395-397 – you are not measuring or considering niche variables, so how your results could suggest anything about niche conservationism? Please, explain better.**

Answer: we decided to expand this line to better explain how our results could suggest niche conservationism (lines 401-403). The lack of dispersal and the restriction to forest habitats could suggest that niche conservationism in this forest clade is important. We hope that now this statement is better explained. Thanks a lot for this comment.

**Figs 3 and 4 – perhaps you could add a square or a lateral bar with the name of forest lanceheads after the names of the focal Bothrops to better highlight this group**

Answer: thanks for the suggestion. We added a green square highlighting the names of the focal group. Thanks again.
