# Supplementary material for: The role of vicariance and dispersal on the temporal range dynamics of forest vipers in the Neotropical region
Source: PLoS One. 2021 Sep 17;16(9):e0257519. doi: 10.1371/journal.pone.0257519 (PMC8448354; doi:10.1371/journal.pone.0257519)
Supplement: Attachment — Submitted filename: Response to Reviewers.docx [file pone.0257519.s013.docx]

**Rebuttal Letter**

We are very thankful for the comments and revisions on our revised manuscript made by the reviewer #2. Below is our response to every point raised by the reviewer to improve our work and to fully meet PLOS ONE’s publication criteria. We hope that we satisfyingly addressed these points and that our manuscript will now be suited for publication.

Sincerely,

On behalf of all authors,

Matheus Pontes-Nogueira (corresponding author).

**Reviewer #2**

**In this new version, Pontes-Nogueira and collaborators have addressed all my comments. The manuscript looks fine, almost ready to be accepted for publication, except for few details in the added information about C. durissus which requires to be more specific and furthermore has a typo. Nevertheless, this is a very minor modification that can be easily solved.**

Answer: thank you for these comments. We made the modifications that you suggested and hope that now the manuscript meets the journal standards. Thanks again.

**Regarding this new information about C. durissus (lines 222-225):**

**First, the information in between () can be deleted because is repeating the information in the sentence, it does not provide any explanation. Second, I think that the authors must say something like “C. durissus is a widespread south American pit viper species showing the highest number of units within its range”. I guess, this is the reason why the authors look for other species out of the Bothrops clade to parameterise models. Finally, "specie" should be change by “species”.**

Answer: We removed the information inside (). We also wrote something similar that you suggested (lines 219 – 226). Thanks for this suggestion because we see that it needed more information about C. durissus. And finally, there was no need for the change in “specie” because we rewrote the entire sentence. Thanks a lot for the suggestions.
